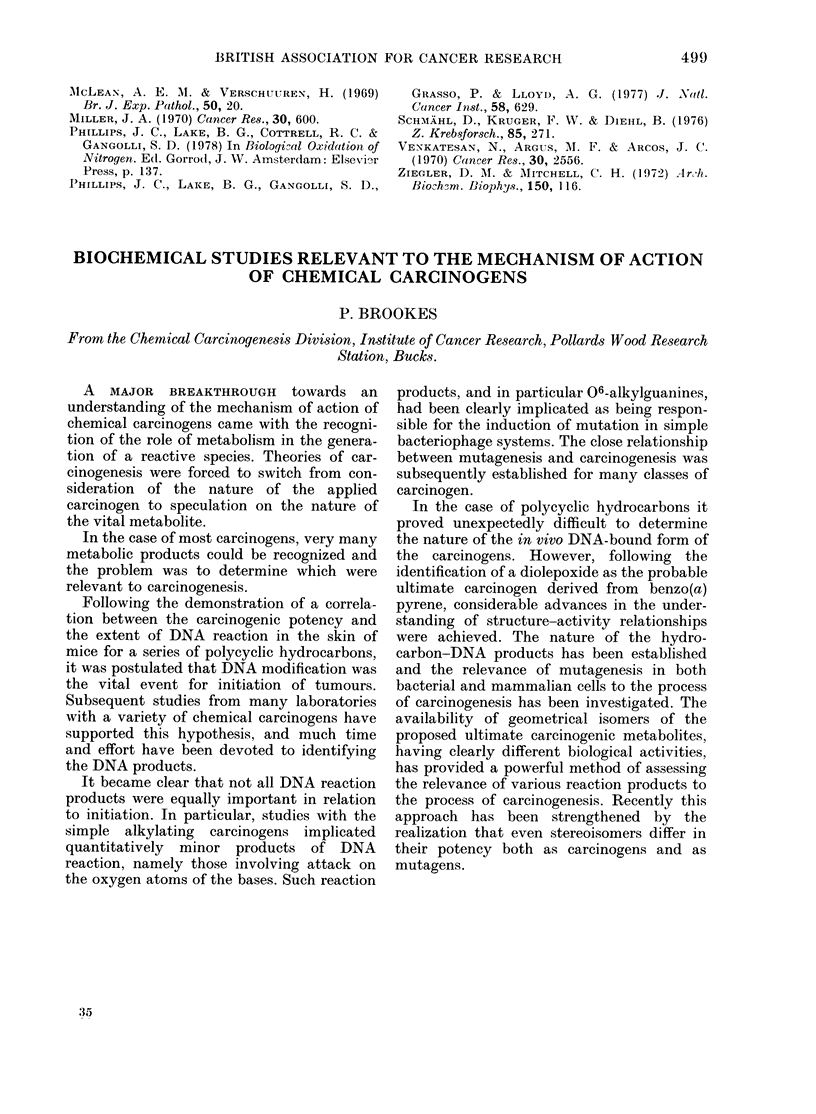# Biochemical studies relevant to the mechanism of action of chemical carcinogens.

**DOI:** 10.1038/bjc.1980.80

**Published:** 1980-03

**Authors:** P. Brookes


### BIOCHEMICAL STUDIES RELEVANT TO THE MECHANISM OF ACTION OF CHEMICAL CARCINOGENS

P. BROOKES

*From the Chemical Carcinogenesis Division, Institute of Cancer Research, Pollards Wood Research Station, Bucks.*

A MAJOR BREAKTHROUGH towards an understanding of the mechanism of action of chemical carcinogens came with the recognition of the role of metabolism in the generation of a reactive species. Theories of carcinogenesis were forced to switch from consideration of the nature of the applied carcinogen to speculation on the nature of the vital metabolite.

In the case of most carcinogens, very many metabolic products could be recognized and the problem was to determine which were relevant to carcinogenesis.

Following the demonstration of a correlation between the carcinogenic potency and the extent of DNA reaction in the skin of mice for a series of polycyclic hydrocarbons, it was postulated that DNA modification was the vital event for initiation of tumours. Subsequent studies from many laboratories with a variety of chemical carcinogens have supported this hypothesis, and much time and effort have been devoted to identifying the DNA products.

It became clear that not all DNA reaction products were equally important in relation to initiation. In particular, studies with the simple alkylating carcinogens implicated quantitatively minor products of DNA reaction, namely those involving attack on the oxygen atoms of the bases. Such reaction products, and in particular $O^6$-alkylguanines, had been clearly implicated as being responsible for the induction of mutation in simple bacteriophage systems. The close relationship between mutagenesis and carcinogenesis was subsequently established for many classes of carcinogen.

In the case of polycyclic hydrocarbons it proved unexpectedly difficult to determine the nature of the *in vivo* DNA-bound form of the carcinogens. However, following the identification of a diolepoxide as the probable ultimate carcinogen derived from benzo(*a*) pyrene, considerable advances in the understanding of structure–activity relationships were achieved. The nature of the hydrocarbon–DNA products has been established and the relevance of mutagenesis in both bacterial and mammalian cells to the process of carcinogenesis has been investigated. The availability of geometrical isomers of the proposed ultimate carcinogenic metabolites, having clearly different biological activities, has provided a powerful method of assessing the relevance of various reaction products to the process of carcinogenesis. Recently this approach has been strengthened by the realization that even stereoisomers differ in their potency both as carcinogens and as mutagens.